# Falling back on technology mindfully during COVID-19 pandemic: NUMed campus experience

**DOI:** 10.15694/mep.2020.000102.1

**Published:** 2020-05-20

**Authors:** Bhavani Veasuvalingam, Micheala Louise Goodson

**Affiliations:** 1 Newcastle University Medicine Malaysia

**Keywords:** Online education, medical education, technology, COVID-19

## Abstract

This article was migrated. The article was marked as recommended.

COVID-19 pandemic has forced technology to evolve as the most challenging means of delivering teaching in addressing the cordon sanitaire. Technology has already been transforming most operation in medical education and secured strong footage in medical schools. NUMed, like any other higher educational institution, has started planning and implementing various strategies to ensure teaching is not hampered during this crisis. ReCaps, online tutorials, discussion boards, online seminars, webinars and quizzes were utilized in reflecting on the current situation. The paper assesses and mitigates the immediate change that has breached the boundaries of students and staffs to be engaged with strategic planning and adoption during the COVID-19 situation. This paper illustrates the interrelatedness and navigates across borders to leverage the possible planning and implementing educational technology as the pedagogical means to ensure the quality of medical education is preserved in fact enhanced through these strategies.

## Introduction

The coronavirus, phylogenetically in the SARS-CoV clade which caused the COVID-19 outbreak in Wuhan, China has changed the landscape of education as a whole and illuminates technology as the sole survivor of the current crisis. In fact, it became the largest online education activity on large-scale and had made learning possible to almost 270 million students and 20 million faculties ( Zhou *et al.,* 2020). On a similar context, medical education has undergone a major distracting change as a result of the COVID-19 contagion and technology has been fundamentally supporting ( Goh and Sandars, 2020) in every way possible. Technology is now at the forefront of change as a great enabler in continuously holding medical education for medical students across the globe.

On 18 ^th^ March 2020, in the light of the novel coronavirus global outbreak, and in the attempt to overcome the spread of the infection, the Prime Minister of Malaysia declared Movement Control Order (MCO) throughout the country (MOH, 2020).

The implementation of lockdown and social distancing to squelch the new coronavirus has resulted in the closure of most medical campuses and home-based working becoming increasingly inevitable (Poh, 2020). Like most institutions, NUMed has its challenges and began planning strategies to ensure the delivery of teaching and learning activities are not compromised in any way. COVID-19 pandemic has resulted in various ways of teaching to be delivered to a virtual platform and accessibility. Considering TEL can be a challenge as well as a barrier for higher education, it is pertinent that the academic environment ( Lytras, Sarirete, and Damiani, 2020) ensure mindful planning and implementation of TEL.

## Purpose

This paper aims to share on the mindful planning and implementation of NUMed medical education during the COVID-19 pandemic. The inoculable disease has heightened everyone’s level of awareness on the use of various available technologies as its mainstream of teaching delivery. The newly revised integrated medical curriculum echoes the contemporary trends of competency-based medical education (CBME) (DE. Powell, 2018). CBME features student-centredness, active engagement, the flexibility of instructional design, and constructive alignment to its learning activities with an assessment. NUMed being the branch campus of Newcastle University, United Kingdom is on the edge of technology support. However, we need to be mindful of using technology to enhance learning and achieve the intended learning outcome and not driven merely by technology ( Dror, Schmidt, and O’Connor, 2011).

## NUMed’s Adoption to Educational Technology

NUMed needed to quickly reform and adapt to the emerging crisis by embracing the available tools and resources. A swift change from face to face to the online mode of teaching had to be planned. The campus-based lectures needed to be converted to online lecture recording, then uploaded in the Virtual Learning Environment (VLE). The online lectures need to be student-centred and promote self-directed learning. This includes videos, listening to records, online discussion activities and engaging in short formative quiz sessions.

## Planning the Online Teaching

The Online Medical Education platform must deliver the core teaching and learning activities of curriculum content with ‘reasonably high filtration” as highlighted below:

1. Converting lecture hours to online lecture recordings via ReCap video recordings.
2. Ensuring the learning outcomes are considered for every module delivered online.
3. Ensuring interactive elements are in place within the one-hour delivery of lectures.
4. Formative assessment to check to understand with the inclusion of quizzes and short tests.
5. To tackle the variability of an internet connection, all works are recorded and uploaded for later viewing.
6. Follow-up of students completed online work and feedback provision accordingly.
7. Identify suitable online tools and resources to suitably facilitate learning and achievement of the specified learning outcome. Example: Discussion board as a platform for interaction and promote critical thinking and problem-solving skills by case-based teaching.
8. Providing guidance and support to faculty to use and develop online teaching materials.
9. Information Technology (IT) department to take measures for the smooth execution of online resources.
10. Promote Library Resources to support online teaching and learning.

## Creating the Online Learning Environment

### Online lectures

NUMed immediately converted face to face lectures with ReCap lecture recordings. Staffs were instructed to facilitate and organize their lecture recordings in teaching spaces utilizing online booking form available on the website. Using the ReCap, personal capture, new recordings were developed as their preferred new standalone recording with a set of slides. This is quickly linked to a module in the Virtual Learning Environment (VLE). VLE is reported to give students more control and accountability towards their own learning and enhances problem-solving skills ( Deshmukh and Pathak, 2020). The adoption of VLE as a single model of teaching delivery potentially create the transformative change ( Goh and Sandars, 2020). NUMed views this as its main pedagogical platform.

### e-Tutorials

Tutorials and seminars made online encourage students to make the most of the opportunities for discussion and debate. Ideally, online tutorials were best to be kept short by chunking lessons to a smaller proportion, considering the cognitive load with quizzes to capture student’s accountability ( Williams and Gil, 2018). E-tutorials were perceived as valuable in reinforcing standard classroom learning methods as it promotes revision at own pace and time ( McGuinness and Fulton, 2019). Options on synchronous online meetings (where all members of the group are online simultaneously) or asynchronous discussions running over a longer period provided.

Faculty needed to ensure the level of engagement made by the students towards these activities. Accessibility to online books, websites, journal articles, audio or video resources were provided to stimulate discussion. Quality of materials developed verified by deans and assistance deans to course structure, flexibility in the process of learning, and technological quality such as accessibility in responding, ease of use are acceptable ( Harsasi and Sutawijaya, 2018).

### Online Seminar

Seminar with webinar toolswas offered as an alternative, that bringsimmediate and personable touch. It mimics the sequence and structure of a normal week and provides a fixed focus to students for engaging with pre-work and a deadline for when pre-work should be completed. Furthermore, options were given for parts or all of the sessions to be student-led with breakout rooms used to set tasks for sub-groups of attendees. There may be unfamiliar technology to use in an educational context for students, both as seminar leaders and participants. To address this, support was provided for all staff and students with access to zoom and microsoft teams for running online webinars made available on PC, Mac, Web and mobile devices.

Collaborative documentswere made available by setting up in Teams using Word, Excel or other tools in Office 365 to enable both synchronous and asynchronous opportunities for working together. It is crucial to ensure not too large numbers of associates engage in a single document, as it can get confusing and hence proper planning is imperative. This kind of joined work functions better with smaller numbers. Microsoft Teams can also support small group collaborations.

### Discussion boards

NUMed Blackboard has built-in discussion boards and this is effectively used for asynchronous discussions, with student and teacher post over a period of time. The asynchronous environment provides opportunities for deeper reflection and easier to include students in different time zones. As it is a text-based approach, it has less chance of technical challenges. Threads can be started by staff or students, involving in frequent and active facilitation from the seminar leader to assist discussions, give informal feedback and encourage interaction.

Challenges in using a discussion board would be the need to manage students’ expectations. That is when and how much they are meant to contribute, and how they should respond to comments from others in the discussion. Setting some basic guidelines for expectations on contributions would be helpful. Students were instructed to collaborate on tasks over a defined period and report back to a discussion space or online seminar. An option for using both synchronous and asynchronous approaches was provided for example asynchronous preparation leading to a real-time timetabled session, or timetabled group sessions leading into asynchronous work reporting into a discussion forum by a specified deadline. Student groups can negotiate how and when to work using recommended tools (Teams/Office 365) or make use of other tools they are familiar with.

### Online formative assessment and feedback

Formative assessment for learning was implemented by quizzes to test students understanding of the module taught module using Blackboard. This is believed to further act as a useful revision exercise for the students. The feedback provision is crucial to enhance and promote learning, further enhancing self-directed learning ( Nagandla, Sulaiha, and Nalliah, 2018). Furthermore, it is less time consuming and this overcomes the major concern dwelled by medical educators in delivering and handling feedback. The test is considered to be delivered by a single best answer (SBA) and short answer question (SAQ) with feedback targeted over a period of time. Staffs are to generate their own online formative assessment methods with the suggestions proposed above, with feedback delivered in a timely manner.

## Faculty Development for Online Support

To support NUMed medical educators on faculty development, a series of webinar session provided on the executions of the available tools and resources. This was supported by Newcastle, University, UK, Faculty of Medical School (FMS), Technology Enhanced Learning (TEL) team. To guide the teaching staff, the FMS TEL team and the University information technology (IT) department developed webpages to further support colleagues to work from home. In addition **,** a daily series of webinars on different tools and approaches to remote delivery including twice-daily online drop-in sessions were offered. The remote and home working toolkit for staff and students with specific guidance regarding remote working was given. The Learning Teaching Development Service (LTDS) developed some good pages to support colleagues transitioning to online teaching.

## Challenges

Landing fully on TEL significantly increases the workload for the faculty as more preparation time is required to ensure learning outcomes are met and adequately prepare students for upcoming assessments. Staffs need to be made aware of clear expectations for students such as what is expected of them and when with explicit indications of how the alternative activity continues to meet the learning outcomes of the module and the programme. Careful planning is needed by the educator in advance of the session materials. Another pint to highlight is to ensure timetabling does not unintentionally collide with live teaching sessions on other modules.

Restructuring the whole educational system needs a comprehensive well-rounded communication and teamwork from every level. In such a short time frame, everyone had to work hard and get the daily operational task moving. Competent support from IT staffs allowed conversion work to take place quickly. Allocation of task and roles communicated clearly to all faculty members. Another significant aspect of technology delivered to a large group of students is that it lacks individual attention as it is delivered in a standard approach ( Sharma, Doberty and Dong, 2017). Being mindful that students varied in their levels of understanding and previous experience depending on modules that they had completed prior to the COVID-19 episode. Considering all the above points, technology should drive learner and enhance learning ( Dror, Schmidt, and O’Connor, 2011).

Thus, we need to ensure the tools and resources used are student-friendly and mindful of the cognitive effect of the tool or resources prepared. This can be regulated by restricting the amount of information delivered, appropriate interaction, engagement and participation of learners as commented by Dror. Other unanticipated challenges included overseas students returning home to countries different time zones which limited delivery of some interactive teaching sessions. Access to online communication and conferencing applications differs around the World and gaining access for all students on one learning platform was not easy to facilitate. For students requiring teaching on practical procedure skills that fulfil the learning outcomes of their current curriculum, a need has been identified to make video recordings locally as available open access online material varies in quality and suitability for General Medical Council (GMC), UK and Malaysian Medical Council skill requirements.

## Implication of the change

The total transformation that has taken place due to the COVID-19 pandemic will probably have a significant impact on medical educators teaching delivery and students learning pattern. The change is viewed to be transformational as it will become well integrated within the social system ( Goh and Sandars, 2020). Equally, once NUMed faculty and staff become comfortable within the new online environment, technology may enhance students learning and achieve the learning outcomes found to be well facilitated by technology. Though not all will be different, this turning point has increased faith in technology sparking a change in behaviour away from traditional approaches (Omian, 2020).

## Take Home Messages

In the midst of this global pandemic, we are reflecting in real-time on how systems undergo a large-scale transformation. Knowledge during crisis management may not be something expected by all, during such time but can be acquired by understanding the priorities and taking strategic measures to collectively embark on the transformation process safely and effectively. The collective support of all members of staff including the administrative staff and students is imperative. Innovative thinking and collaborative working groups have made this a successful endeavour.

## Notes On Contributors

**Bhavani Veasuvalingam**, is a Senior Lecturer for Medical Education, Newcastle University Medicine Malaysia, Johor Baru Campus. Medical educationalist, local program director of Post Graduate Certificate in Medical Education, Newcastle UK. Lead for Faculty Development Program and Pedagogical Research Projects. The main author, conceptualized, wrote, and revised manuscript based on comments and suggestions from the other author.

**Dr. Micheala Louise Goodson**, Dean of Research, Newcastle University Medicine Malaysia, Johor Baru Campus. Reviewed manuscript.
