# Supplementary material for: Falling back on technology mindfully during COVID-19 pandemic: NUMed campus experience
Source: MedEdPublish (2016). 2020 May 20;9:102. [Version 1] doi: 10.15694/mep.2020.000102.1 (PMC10712600; doi:10.15694/mep.2020.000102.1)
Supplement: Peer review history

# Reviewer report: Reviewer response for version 1

**DOI:** 10.21956/mep.20043.r30693

**Published:** 2020-06-04

**Authors:** sathyanarayanan varadarajan

**Affiliations:** 1 SRM Medical College, SRM University

This review has been migrated. The reviewer awarded 4 stars out of 5 This interesting article illustrates the experience of mindful planning and implementing various strategies of educational technology in a medical school during the challenging COVID-19 pandemic. These strategies include online lectures, e-Tutorials, online Seminar, discussion boards and online formative assessment and feedback. The authors also highlight upon the challenges faced during the implementation of these strategies and how they overcome the challenges with mindful planning. They conclude that, they have made this as a successful endeavour because of innovative thinking and collaborative working groups. This article provides us a good example of showing us that even a crisis can be transformed if we can plan together creatively and implement mindfully . This case study will be helpful to all the medical educators who are involved in planning and implementing various strategies of medical educational technology to provide high quality of medical education in this difficult time.

Reviewer Expertise:

NA

No decision status is available

# Reviewer report: Reviewer response for version 1

**DOI:** 10.21956/mep.20043.r30694

**Published:** 2020-05-20

**Authors:** P Ravi Shankar

**Affiliations:** 1 American International Medical University

This review has been migrated. The reviewer awarded 4 stars out of 5 This is another in a series of papers dealing with the response of medical schools to the COVID-19 pandemic. In this article the authors provide an overview of different modifications made at their university during the shift to online learning. The main approach adopted by the university is similar to that described in other manuscripts. In certain areas I found reading the manuscript was a bit difficult. The authors can define TEL as technology enhanced learning on first use in the Introduction section. I would also be interested in knowing about whether any specific challenges were faced in shifting to online learning in the Malaysian context. The institution had the benefit of support from a well-established institution in the United Kingdom. The institution also has international students. Some information about possible challenges faced by them in participating in online learning will be useful.

Reviewer Expertise:

NA

No decision status is available
